# ﻿Five new species of *Cortinarius* (Cortinariaceae) from Yunnan, China, based on molecular and morphological evidence

**DOI:** 10.3897/mycokeys.116.146710

**Published:** 2025-04-14

**Authors:** Liu-Kun Jia, Zi-Rui Wang, Zhu-Liang Yang

**Affiliations:** 1 State Key Laboratory of Phytochemistry and Natural Medicines, Kunming Institute of Botany, Chinese Academy of Sciences, Kunming, 650201, China; 2 Yunnan Key Laboratory for Fungal Diversity and Green Development, Kunming Institute of Botany, Chinese Academy of Sciences, Kunming, 650201, China; 3 College of Life Sciences, University of Chinese Academy of Sciences, Beijing, 101408, China; 4 School of Ecology and Environmental Science, Yunnan University, Kunming, 650500, China

**Keywords:** Diversity, morphology, new taxa, phylogeny, taxonomy

## Abstract

*Cortinarius* is a globally distributed, exceptionally species-rich genus of Cortinariaceae, serving as important ectomycorrhizal fungi. Yunnan province, located in southwestern China, boasts a vast array of environmental conditions and fungal resources, with numerous new *Cortinarius* species yet to be discovered. Based on morphological evidence and phylogenetic inference using a two-locus dataset, five novel species have been identified within the genus, namely *C.brunneoverrucosus*, *C.coriaceus*, *C.fuscocandidus*, *C.neodisjungendus*, and *C.sinoconfirmatus*. Notably, two of these species (*C.brunneoverrucosus* and *C.neodisjungendus*) occur in subtropical areas, while the other three species (*C.coriaceus*, *C.fuscocandidus*, and *C.sinoconfirmatus*) inhabit subalpine temperate areas. Taxonomic descriptions for these five species are provided.

## ﻿Introduction

*Cortinarius* (Pers.) Gray, belonging to the order Agaricales, is the most species-rich genus within the family Cortinariaceae. Currently, over 2,000 species have been formally described (Liimatainen et al. 2022). It is widely distributed across tropical to subpolar areas in both the Northern and Southern Hemispheres, holding irreplaceable ecological, research, and economic value (Kirk et al. 2008; Soop et al. 2019; Liimatainen et al. 2020, 2022). Based on recent genomic and multi-locus sequence data, *Cortinarius* sensu lato has been split into ten genera, with the core groups within *Cortinarius* s. l. being transferred to *Cortinarius* sensu stricto, emended in Liimatainen et al. (2022).

*Cortinarius* s.s., typified by *C.violaceus* (L.) Gray, has several distinguishing features. These include a pileus adorned with fibrillose squamules, a fibrillose cortina, a negative KOH reaction, and rusty brown basidiospores with weakly to strongly verrucose ornamentations. Additionally, it features a duplex pileipellis with a hypoderm that is variably developed. The basidiomata vary widely in size, ranging from very small to large, and can be dry to glutinous in texture. They exhibit a diverse array of colors, with brown being the most common (Bidaud et al. 2000, 2012; Liimatainen et al. 2015, 2017, 2020, 2022; Soop et al. 2019; Ammirati et al. 2021; Zhou et al. 2023; Long et al. 2024). Liimatainen et al. (2022) identified 11 recognized subgenera under Cortinarius s.s., including subgen. Cortinarius, subgen. Dermocybe (Fr.) Trog, subgen. Illumini Liimat., Niskanen & Kytöv, subgen. Leprocybe M.M. Moser, subgen. Iodolentes Niskanen & Liimat., subgen. Orellani (M.M. Moser) Gasparini, subgen. Telamonia (Fr.) Trog, subgen. Infracti Niskanen & Liimat, subgen. Camphorati Liimat., Niskanen & Ammirati, subgen. Myxacium (Fr.) Trog, and Paramyxacium M.M. Moser & E. Horak.

The research on the genus *Cortinarius* primarily originated and has remained concentrated in Europe, North America, and Oceania (Peintner et al. 2002a, 2002b, 2004; Garnica et al. 2003, 2005; Liimatainen et al. 2014, 2015, 2017, 2020, 2022; Niskanen 2014, 2020; Soop et al. 2019), whereas studies in East Asia are still insufficient. Since the reporting of *C.testaceus* Cooke in China by Teng and Ou (1937), numerous Chinese mycologists have described *Cortinarius* species across various areas, including northeast, north, east, south, and southwestern parts of China (Deng 1963; Li 1980; Mao and Zong 1988; Ying and Zang 1994; Zang 1996; Mao 2000, 2009). However, most of these species’ names recorded by the aforementioned studies are based on those from Europe, North America, and Oceania, and their distinctiveness in China awaits confirmation through molecular evidence. Recently, based on a combination of morphological and molecular systematic evidence, 26 new *Cortinarius* species from China have been published (Wei and Yao 2013; Xie et al. 2019, 2020, 2021a, 2021b, 2022, 2023; Yuan et al. 2020; Luo and Bau 2021; Zhang et al. 2023; Zhou et al. 2023; Long et al. 2024), indicating that the species diversity of *Cortinarius* in China is high, and potential undiscovered species may exist within the genus.

In this study, five *Cortinarius* species new to science were identified in Yunnan, southwestern China. Based on a combination of morphological observations and phylogenetic analysis, we provide descriptions of these species.

## ﻿Materials and methods

### ﻿Specimens and morphological description

Macro-morphological characteristics were described based on fresh basidiomata, detailed field notes, and photographs taken in situ. Colors in the descriptions were coded following Kornerup and Wanscher (1981). The basidiomata size, determined by pileus width, was categorized as tiny (< 1.5 cm), small (1.5–3 cm), medium-sized (3–5 cm), or large (> 5 cm). Additionally, ‘L’ refers to the number of lamellae reaching the stipe, while ‘l’ denotes the number of lamellulae located between two lamellae.

Microscopic structures were observed with light microscopy under a ZEISS Axiostar Plus microscope. Dried specimens were sectioned and mounted in a 5% KOH solution or Melzer’s reagent. Congo Red staining was applied when necessary. For observing basidiospore ornamentations, small hymenophoral fragments were taken from dried specimens, mounted on aluminum stubs with double-sided adhesive tape, coated with gold-palladium, and then observed under a ZEISS Sigma 300 scanning electron microscope (SEM) at the Kunming Institute of Botany, Chinese Academy of Sciences.

In the descriptions of basidiospores, the abbreviation [n/m/p] indicates that ‘n’ basidiospores were measured from ‘m’ basidiomata of ‘p’ collections. Dimensions are presented in the form (a–)b–c(–d), where the range ‘b–c’ includes a minimum of 90% of the measured values, with extreme values “a” or ‘d’ given in parentheses. The ratio of basidiospore length to width in side view is represented by Q. The mean values and average Q of basidiospores, along with standard deviations, are indicated as “av.” and ‘Qav.’, respectively. Basidiospore shapes were determined based on descriptions by Bas (1969) and Kirk et al. (2008).

The studied collections were deposited in the Cryptogamic Herbarium of Kunming Institute of Botany, Chinese Academy of Sciences (KUN-HKAS).

### ﻿DNA extraction, polymerase chain reaction (PCR), and sequencing

Total genomic DNA was extracted from dried specimens using an Ezup Column Fungi Genomic DNA Purification Kit (Sangon Biotech, Shanghai, China). The ITS region was amplified using the primers ITS1F/ITS4. For older specimens, primer combinations ITS1F/ITS2 and ITS3/ITS4 were also employed (White et al. 1990; Gardes and Bruns 1993). The ribosomal large subunit 28S region (nrLSU) was amplified using the primers LROR/LR5 (Vilgalys and Hester 1990; Hopple and Vilgalys 1994).

PCR reactions were conducted using an ABI 2720 Thermal Cycler, Veriti^TM^ Dx 96-Well Thermal Cycler, or SimpliAmp^TM^ Thermal Cycler (Applied Biosystems, Foster City, CA, USA). The PCR settings for the ITS1F/ITS4 were 94 °C for 5 min, followed by 35 cycles of 94 °C for 40 s, 52 °C for 40 s, and 72 °C for 1 min, with a final extension at 72 °C for 8 min. For the LROR/LR5 primers, the settings were 94 °C for 5 min, followed by 35 cycles of 94 °C for 40 s, 50 °C for 40 s, and 72 °C for 1 min, with a final extension at 72 °C for 8 min.

The PCR products were purified using a Gel Extraction and PCR Purification Combo Kit (Spin-column) (Bioteke, Beijing, China). After purification, the products were sequenced on an ABI-3730-XL DNA Analyzer (Applied Biosystems, Foster City, CA, USA) using the same primer combinations as those used for the PCR.

### ﻿Phylogenetic analysis

Forward and reverse sequences were assembled and edited with SeqMan (DNA STAR package; DNAStar Inc., Madison, WI, USA). The ITS sequences were used to infer related taxa through a BLASTn search in GenBank (https://blast.ncbi.nlm.nih.gov/Blast.cgi). The top hits in the BLASTn results confirmed that our specimens belonged to the genus *Cortinarius*. Related species were selected for the phylogenetic analyses based on BLASTn results (> 90% identity) and references from publications by Soop et al. (2019), Liimatainen et al. (2017, 2020), and Ammirati et al. (2021). A total of 64 collections representing 57 species were included in this study, with five species from the sect. Leprocybe selected as outgroups.

Alignments were constructed using MAFFT v7.3.10 (Katoh and Standley 2013) and optimized using BioEdit v7.2.5 (Hall 1999). The final alignments have been submitted to TreeBASE (http://purl.org/phylo/treebase/phylows/study/TB2:S31924).

Phylogenetic analyses were conducted using maximum likelihood (ML) and Bayesian inference (BI) methods, implemented in IQ-TREE v2.2.0 (Nguyen et al. 2015) and MrBayes 3.2.7 (Ronquist et al. 2012). The best-fit substitution model for ML analyses using the ITS+nrLSU matrix was determined with the ‘-MFP’ option in IQ-TREE v2.2.0 (Kalyaanamoorthy et al. 2017) based on the Akaike Information Criterion (AIC) (Table 1). For ML analyses, 1,000 replicates of the Shimodaira-Hasegawa-like aLRT test (SH-aLRT) (Guindon et al. 2010) and 1,000 replicates of the ultrafast bootstrap (UFB) (Hoang et al. 2018) were set. The ‘-p’ option was used for reading the partition file (ITS: 1–744; nrLSU: 745–1627), while other parameters remained at default settings. For BI analyses, substitution models were selected based on IQ-TREE v2.2.0 outputs (Table 1). Four Markov chains were run twice from random starting trees for 10 million generations, with sampling every 100^th^ generation. The stop value (stopval) was set to 0.001. Parameters and sampled trees were summarized after discarding the first 25% of trees as burn-in using the ‘sump’ and ‘sumt’ commands in MrBayes 3.2.7.

**Table 1.** DNA substitution models selected for phylogenetic analysis based on the ITS-nrLSU matrix.

Loci	Models for maximum likelihood (IQ-TREE)	Models for Bayesian inference (MrBayes)
ITS	TIM2+F+I+R2	lset applyto = (ITS) nst = 6 rates = Invgamma
nrLSU	TN+F+I	lset applyto = (nrLSU) nst = 2 rates = Propinv prset applyto = (all) statefreqpr = Dirichlet(1,1,1,1)

## ﻿Results

### ﻿Molecular analyses

**Figure 1.**
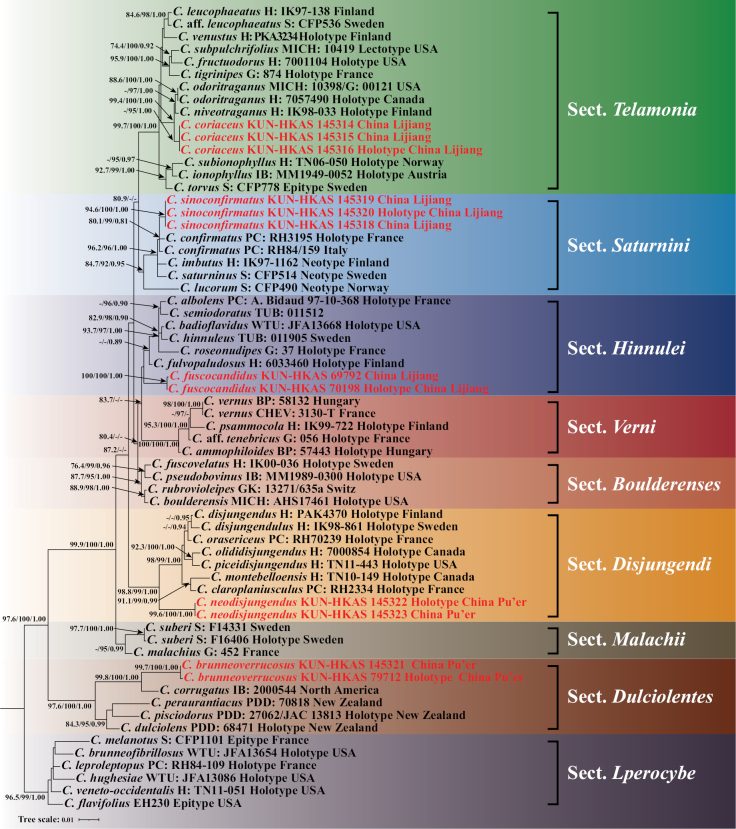
Maximum-likelihood phylogenetic tree of *Cortinarius* inferred from the concatenated ITS-nrLSU matrix. SH-aLRT support values ≥ 80%, UFB values ≥ 90% for ML, and BPP values ≥ 0.80 for BI are shown above the nodes as SH-aLRT/UFB/BPP. Sequences generated in this study are highlighted in red.

The dataset comprised a total of 78 sequences, including 24 newly generated and 54 downloaded sequences (64 ITS, 14 nrLSU) from 64 collections representing 57 species (Table 2). The concatenated dataset (ITS-nrLSU) consisted of 1,627 positions after excluding poorly aligned regions. Accession numbers for all the sequences used for molecular analyses are provided in Table 2. Both ML and BI trees exhibited the same topology; therefore, only the ML tree, with the SH-aLRT support values, UFB values, and Bayesian posterior probabilities (BPP), is shown (Fig. 1).

**Table 2.** Voucher information, GenBank accession numbers of the samples used in the phylogenetic analysis.

Taxa	voucher	Status	Locations	Section	GenBank Accession No.		Sequence origin
					ITS	nrLSU	
Cortinariusaff.leucophaeatus	S: CFP536		Sweden	*Telamonia*	KC608590	in ITS	Liimatainen et al. 2020
C.aff.tenebricus	G: 056		France	*Verni*	MT935483	-	Liimatainen et al. 2020
*C.albolens*	PC: A. Bidaud 97-10-368	Holotype	France	*Hinnulei*	MT934855	in ITS	Liimatainen et al. 2020
*C.ammophiloides*	BP: 57443	Holotype	Hungary	*Verni*	NR_171309	-	Liimatainen et al. 2020
*C.badioflavidus*	WTU: JFA13668	Holotype	USA	*Hinnulei*	KU041723	in ITS	Liimatainen et al. 2020
*C.boulderensis*	MICH: AHS17461	Holotype	USA	*Boulderenses*	DQ499466	in ITS	Liimatainen et al. 2020
*C.brunneofibrillosus*	WTU: JFA13654	Holotype	USA	*Leprocybe*	MW009188	-	Ammirati et al. 2021
***C.brunneoverrucosus***	**KUN-HKAS 79712**	**Holotype**	**China**	***Dulciolentes***	**PQ772212**	**PQ772224**	**This study**
***C.brunneoverrucosus***	**KUN-HKAS 145321**		**China**	***Dulciolentes***	**PQ772211**	**PQ772223**	**This study**
*C.claroplaniusculus*	PC: RH2334	Holotype	France	*Disjungendi*	NR_131844	-	Liimatainen et al. 2020
*C.confirmatus*	PC: RH84/159		Italy	*Saturnini*	KX964440	in ITS	Liimatainen et al. 2017
*C.confirmatus*	PC: RH3195	Holotype	France	*Saturnini*	KX964438	in ITS	Liimatainen et al. 2017
***C.coriaceus***	**KUN-HKAS 145314**		**China**	***Telamonia***	**PQ772201**	**PQ772213**	**This study**
***C.coriaceus***	**KUN-HKAS 145315**		**China**	***Telamonia***	**PQ772203**	**PQ772215**	**This study**
***C.coriaceus***	**KUN-HKAS 145316**	**Holotype**	**China**	***Telamonia***	**PQ772202**	**PQ772214**	**This study**
*C.corrugatus*	IB: 2000544		North America	*Dulciolentens*	AF325611	in ITS	Soop et al. 2019
*C.disjungendulus*	H: IK98-861	Holotype	Sweden	*Disjungendi*	NR_131838	-	Liimatainen et al. 2020
*C.disjungendus*	H: PAK4370	Holotype	Finland	*Disjungendi*	KP013190	in ITS	Liimatainen et al. 2020
*C.dulciolens*	PDD: 68471	Holotype	New Zealand	*Dulciolentens*	NR_157914	-	Soop et al. 2019
*C.flavifolius*	EH230	Epitype	USA	*Leprocybe*	MW009217	in ITS	Ammirati et al. 2021
*C.fructuodorus*	H: 7001104	Holotype	USA	*Telamonia*	NR_131827	in ITS	Liimatainen et al. 2020
*C.fulvopaludosus*	H: 6033460	Holotype	Finland	*Hinnulei*	MG136823	in ITS	Liimatainen et al. 2020
***C.fuscocandidus***	**KUN-HKAS 69792**		**China**	***Hinnulei***	**PQ772209**	**PQ772221**	**This study**
***C.fuscocandidus***	**KUN-HKAS 70198**	**Holotype**	**China**	***Hinnulei***	**PQ772210**	**PQ772222**	**This study**
*C.fuscovelatus*	H: IK00-036	Holotype	Sweden	*Boulderenses*	NR_131888	-	Liimatainen et al. 2020
*C.hinnuleus*	TUB: 011905		Sweden	*Hinnulei*	AY669667	in ITS	Liimatainen et al. 2020
*C.hughesiae*	WTU: JFA13086	Holotype	USA	*Leprocybe*	MW009224	in ITS	Ammirati et al. 2021
*C.imbutus*	H: IK97-1162	Neotype	Finland	*Saturnini*	KX964498	in ITS	Liimatainen et al. 2017
*C.ionophyllus*	IB: MM1949-0052	Holotype	Austria	*Telamonia*	MT935168	-	Liimatainen et al. 2020
*C.leproleptopus*	PC: RH84-109	Holotype	France	*Leprocybe*	MW009226	in ITS	Ammirati et al. 2021
*C.leucophaeatus*	H: IK97-138		Finland	*Telamonia*	MT935196	in ITS	Liimatainen et al. 2020
*C.lucorum*	S: CFP490	Neotype	Norway	*Saturnini*	KX964585	in ITS	Liimatainen et al. 2017
*C.malachius*	G: 452		France	*Malachii*	MT934962	-	Liimatainen et al. 2020
*C.melanotus*	S: CFP1101	Epitype	France	*Leprocybe*	MW009230	-	Ammirati et al. 2021
*C.montebelloensis*	H: TN10-149	Holotype	Canada	*Disjungendi*	KP114459	in ITS	Liimatainen et al. 2020
***C.neodisjungendus***	**KUN-HKAS 145322**	**Holotype**	**China**	***Cinnabarini***	**PQ772207**	**PQ772219**	**This study**
***C.neodisjungendus***	**KUN-HKAS 145323**		**China**	***Cinnabarini***	**PQ772208**	**PQ772220**	**This study**
*C.niveotraganus*	H: IK98-033	Holotype	Finland	*Telamonia*	NR_131842	-	Liimatainen et al. 2020
*C.odoritraganus*	H: 7057490	Holotype	Canada	*Telamonia*	MT112154	-	Liimatainen et al. 2020
*C.odoritraganus*	MICH: 10398/G: 00121		USA	*Telamonia*	NR_170852	MK277857	Liimatainen et al. 2020
*C.olididisjungendus*	H: 7000854	Holotype	Canada	*Disjungendi*	NR_131839	-	Liimatainen et al. 2020
*C.orasericeus*	PC: RH70239	Holotype	France	*Disjungendi*	KP013203	in ITS	Liimatainen et al. 2020
*C.peraurantiacus*	PDD: 70818		New Zealand	*Dulciolentens*	KC520543	in ITS	Soop et al. 2019
*C.piceidisjungendus*	H: TN11-443	Holotype	USA	*Disjungendi*	NR_131840	-	Liimatainen et al. 2020
*C.pisciodorus*	PDD: 27062/JAC 13813	Holotype	New Zealand	*Dulciolentens*	MN492664	MH108417	Soop et al. 2019
*C.psammocola*	H: IK99-722	Holotype	Finland	*Verni*	MG136821	-	Liimatainen et al. 2020
*C.pseudobovinus*	IB: MM1989-0300	Holotype	USA	*Boulderenses*	DQ499465	in ITS	Liimatainen et al. 2020
*C.roseonudipes*	G: 37	Holotype	France	*Hinnulei*	MT935391	-	Liimatainen et al. 2020
*C.rubrovioleipes*	GK: 13271/635a		Switz	*Boulderenses*	MT934924	in ITS	Liimatainen et al. 2020
*C.saturninus*	S: CFP514	Neotype	Sweden	*Saturnini*	KX964584	in ITS	Liimatainen et al. 2017
*C.semiodoratus*	TUB: 011512		-	*Hinnulei*	AY669665	in ITS	Liimatainen et al. 2020
***C.sinoconfirmatus***	**KUN-HKAS 145318**		**China**	***Saturnini***	**PQ772206**	**PQ772218**	**This study**
***C.sinoconfirmatus***	**KUN-HKAS 145319**		**China**	***Saturnini***	**PQ772204**	**PQ772216**	**This study**
***C.sinoconfirmatus***	**KUN-HKAS 145320**	**Holotype**	**China**	***Saturnini***	**PQ772205**	**PQ772217**	**This study**
*C.suberi*	S: F16406	Holotype	Sweden	*Malachii*	MT935480	-	Liimatainen et al. 2020
*C.suberi*	S: F14331		Sweden	*Malachii*	MT934927	in ITS	Liimatainen et al. 2020
*C.subionophyllus*	H: TN06-050	Holotype	Norway	*Telamonia*	MF379634	-	Liimatainen et al. 2020
*C.subpulchrifolius*	MICH: 10419	Lectotype	USA	*Telamonia*	NR_170855	in ITS	Liimatainen et al. 2020
*C.tigrinipes*	G: 874	Holotype	France	*Telamonia*	MT935549	in ITS	Liimatainen et al. 2020
*C.torvus*	S: CFP778	Epitype	Sweden	*Telamonia*	MT935556	in ITS	Liimatainen et al. 2020
*C.veneto-occidentalis*	H: TN11-051	Holotype	USA	*Leprocybe*	MW009243	in ITS	Ammirati et al. 2021
*C.vernus*	BP:58132		Hungary	*Verni*	MT935033	in ITS	Liimatainen et al. 2020
*C.vernus*	CHEV 3130-T		France	*Verni*	FN429003	-	Suárez-Santiago et al. 2009
*C.venustus*	H: PAK3234	Holotype	Finland	*Telamonia*	MT935132	in ITS	Liimatainen et al. 2020

Newly generated sequences were marked in bold.

### ﻿Taxonomy

#### ﻿ Cortinarius brunneoverrucosus

Taxon classificationFungiAgaricalesCortinariaceae

Zhu L. Yang, Liu K. Jia & Zi R. Wang sp. nov.

560AADC3-9B45-5E63-925B-E6832883528E

 857350

Fig. 2

##### Etymology.

The epithet “*brunneoverrucosus*” (Lat.) refers to the pileus with brown verrucose squamules of this species.

**Figure 2.**
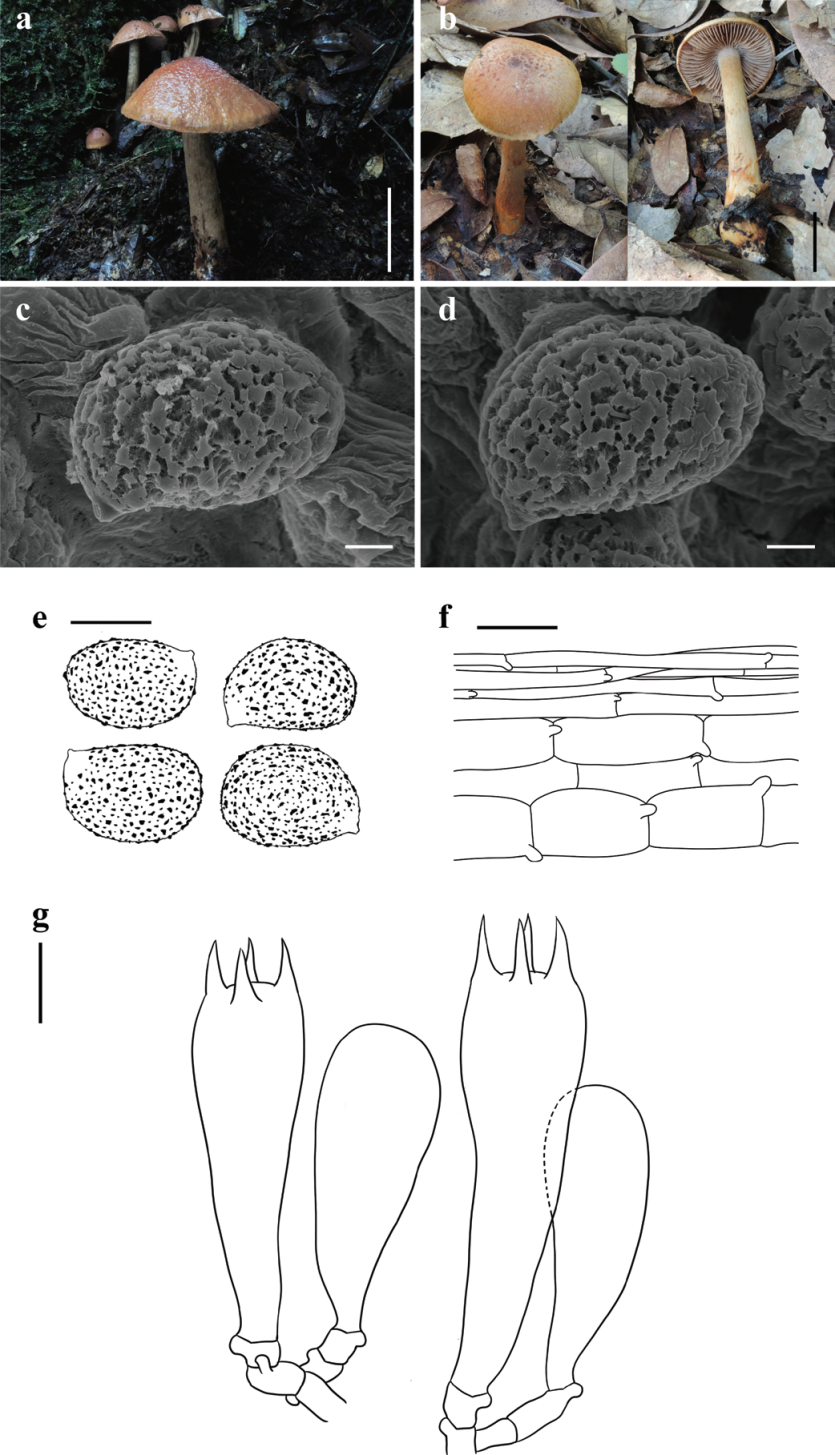
*Cortinariusbrunneoverrucosus* (**a, c–g**KUN-HKAS 79712, Holotype **b**KUN-HKAS 145321) **a, b** basidiomata **c–e** basidiospores **f** pileipellis **g** basidia; and marginal sterile cells. Scale bars: 5 cm (**a, b**); 2 μm (**c, d**); 10 μm (**e**); 20 μm (**f, g**).

##### Holotype.

China • Yunnan Province: Pu’er City, Jingdong Yi Autonomous County Ailao Mountain Subtropical Forest Ecosystem Research Station, Chinese Academy of Sciences, in a subtropical broad-leaved forest with trees of *Lithocarpus*, 24°32.57'N, 101°1.62'E, elevation 2,491 m, 23 July 2013, Yang-Yang Cui 32 (KUN-HKAS 79712). GenBank: ITS: PQ772212, nrLSU: PQ772224.

##### Diagnosis.

*Cortinariusbrunneoverrucosus* is sister to *C.corrugatus* Peck but differs by its yellowish brown to brown pileus with brown verrucose squamules, more robust stipe, relatively wider basidiospores, and exclusive occurrence in subtropical broad-leaved forest with trees of *Lithocarpus* and *Quercus* (Peck 1872; Phillips 2010; Kuo 2020).

##### Description.

***Basidioma*** large. ***Pileus*** 8–10.5 cm diam, hemispherical, viscid, verrucose; yellow-brown to brown (5B7–5C7), darker towards the center (5D8), paler towards the margin (5B3–5B5); covered with brown (5C7) to dark brown (5D8–5E8) verrucose to floccose squamules; margin with innate radial stripes, occasionally with pale yellow (4A2) floccose squamules; context of pileus white (1A1). ***Lamellae*** adnate with decurrent tooth, crowded (L = 64–73, l = 33–38), pale brown (6A2–6A4) with a faint pale pinkish (12A2) tint. ***Stipe*** 8.5–18 × 1.2–2 cm, tapering upwards, pale brown (6A2–6A4) to pale yellow (3A2–3A4), covered with brown (6C4) to orange-brown (5A8) fibrillose squamules; context of stipe white (1A1); basal mycelium white (1A1) with a faint pale pinkish (12A2) tint.

***Basidiospores*** [60/2/2] (12.5–)15–16.5(–17.5) × (10–)11.5–12.5(–15) μm, Q = 1.2–1.5(–1.75), av. = 15.64 ± 1.61 × 12.31 ± 1.48 μm, Qav. = 1.27 ± 0.12, broadly ellipsoid to broadly amygdaliform, strongly verrucose, inamyloid. ***Basidia*** 37.5–50 × 7.5–10 μm, 4-spored, clavate. ***Trama of lamellae*** regular, composed of colorless to yellowish, smooth hyphae 10–12.5 μm wide. ***Cystidia*** absent. ***Pileipellis*** duplex: epicutis weakly developed, 12–15 μm thick, composed of only 3–5 layers of interwoven to parallel, colorless to yellowish, smooth, thin-walled, long-celled hyphae 2.5–4 µm wide; hypocutis composed of parallel, colorless to yellowish brown, cylindrical, thin-walled hyphae 12.5–20 μm wide. ***Clamp connections*** common in all parts of basidioma.

##### Habitat/host.

Summer to autumn. Solitary on soil in subtropical broad-leaved forests with trees of Fagaceae.

##### Distribution.

Currently known from southwestern China.

##### Additional specimen examined.

China • Yunnan Province: Pu’er City, Jingdong Yi Autonomous County, Ailao Mountain Subtropical Forest Ecosystem Research Station, Chinese Academy of Sciences, in a subtropical broad-leaved forest with trees of *Quercus*, 24°32.57'N, 101°1.62'E, elevation 2,424 m, 8 October 2021, Jian-Wei Liu 2440 (KUN-HKAS 145321).

##### Notes.

*Cortinariusbrunneoverrucosus* is characterized by its hemispherical, viscid, verrucose pileus, pale brown lamellae with a slightly pale pinkish tint, and relatively larger, broadly ellipsoid to ellipsoid basidiospores.

*Cortinariusbrunneoverrucosus* is sister to *C.corrugatus* Peck, originally described from the highlands in the United States, under *Aalmialatifolia*, but *C.brunneoverrucosus* is only found in subtropical China, under trees of *Lithocarpus* or *Quercus*. Moreover, *C.corrugatus* differs from *C.brunneoverrucosus* by its convex to broadly convex pileus with distinctively corrugated-wrinkled, thinner stipe, amygdaliform, relatively narrower basidiospores (12–15 × 8–10 μm) (Peck 1872; Phillips 2010; Kuo 2020).

*Cortinariusbrunneoverrucosus* belongs to sect. Dulciolentes Soop, a small section that has previously included only seven species, mainly distributed in Australia, inhabiting forests with Fagaceae, Nothofagaceae, and Myrtaceae (Soop et al. 2019). However, excluding *C.corrugatus*, which is from North America and is agaricoid, as mentioned earlier, three other species from Oceania, *C.peraurantiacus* Peintner & M.M. Moser, *C.pisciodorus* (E. Horak) Peintner & M.M. Moser, and *C.dulciolens* E. Horak, M.M. Moser, Peintner & Vilgalys, are all sequestrate (Moser 1983; Peintner et al. 2002a, 2002b; Soop et al. 2019). The discovery of *C.brunneoverrucosus* represents the first species of sect. Dulciolentes in China and the second agaricoid taxon within the section.

#### ﻿ Cortinarius coriaceus

Taxon classificationFungiAgaricalesCortinariaceae

Zhu L. Yang, Liu K. Jia & Zi R. Wang sp. nov.

C86A92F1-DC06-5C6F-B59F-B8425242CF02

 857351

Fig. 3

##### Etymology.

The epithet “*coriaceus*” (Lat.) refers to the brown pileus with a leathery texture of this species.

**Figure 3.**
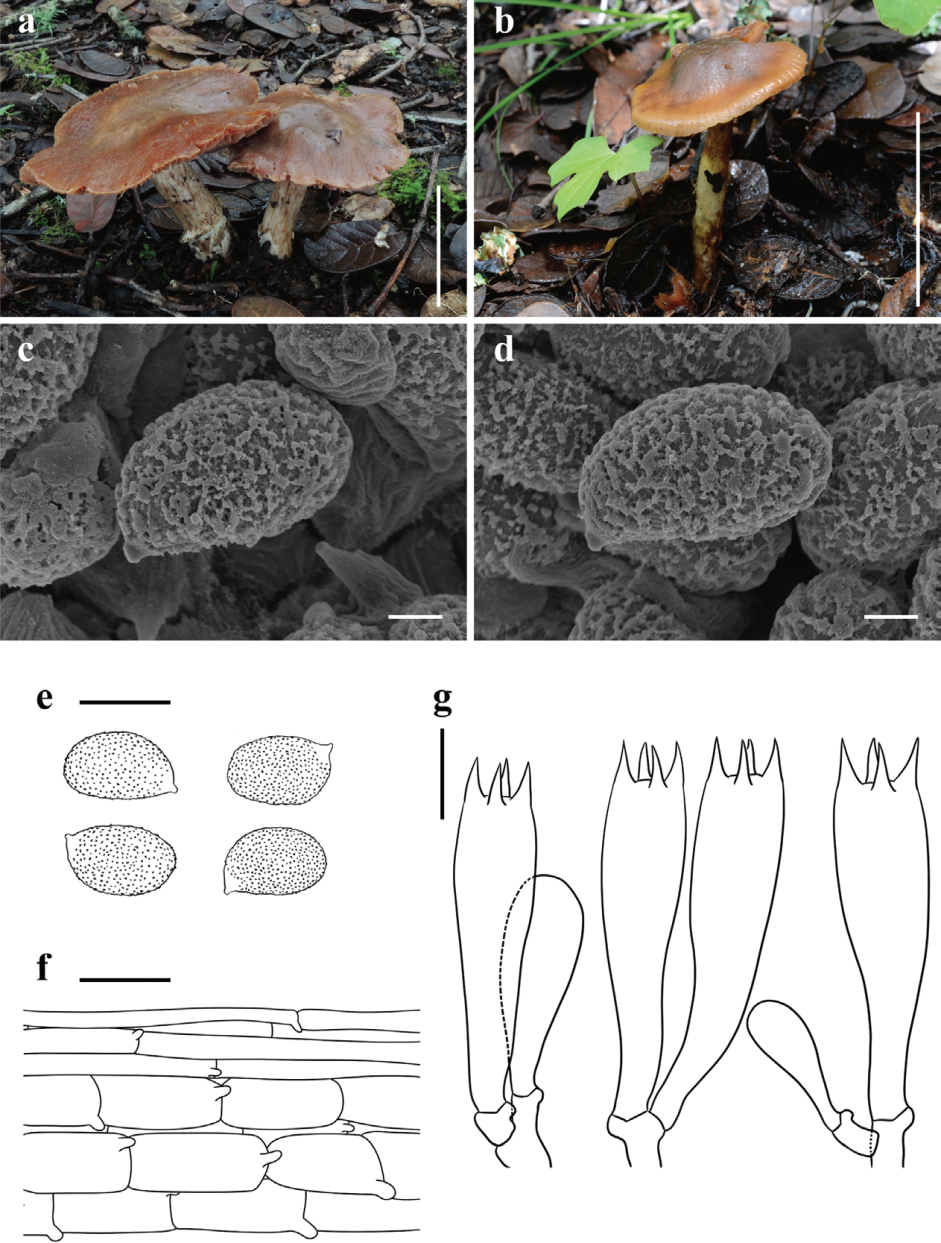
*Cortinariuscoriaceus* (**a, c–g**KUN-HKAS 145136, Holotype **b**KUN-HKAS 145314) **a, b** basidiomata **c–e** basidiospores **f** pileipellis **g** basidia; and marginal sterile cells. Scale bars: 5 cm (**a, b**); 2 μm (**c, d**); 10 μm (**e**); 20 μm (**f, g**).

##### Holotype.

China • Yunnan Province: Lijiang City, Yulong Naxi Autonomous County, Lijiang Alpine Botanical Garden, in a subalpine temperate broad-leaved and coniferous mixed forest with trees of *Quercus* and *Pinus*, 27°0.21'N, 100°10.71'E, elevation 3,340 m, 7 August 2023, Dong-Mei Li 299 (KUN-HKAS 145316). GenBank: ITS: PQ772202, nrLSU: PQ772214.

##### Diagnosis.

*Cortinariuscoriaceus* looks like *C.odoritraganus* Niskanen, Liimat. & Ammirati, but differs in its emarginate lamellae, cylindrical stipe, and relatively larger basidiospores (Niskanen 2020).

##### Description.

***Basidioma*** medium-sized to large. ***Pileus*** 3 cm diam when young, 4.5–7 cm diam when mature, initially slightly campanulate, becoming plano-convex, occasionally with slightly subumbonate center, viscid, with a leathery texture; brown (6C4–6C7), paler (6A2–6A4) towards the center, covered with white (1A1) fibrillose squamules when young; pale brown to brown (6A4–6C4), pale brown (6A2), or dark brown (6D4–6D6) towards the center when mature; margin incurved, with innate radial brownish (6C2–6C3) stripes when young; context of pileus pale brown to brown (6A3–6B3, 6C6). ***Lamellae*** emarginate, medium-spaced (L = 38–52, l = 27–36), pale brown (6A4) with a faint pinkish (12A2) tint when young, later brown (6C4–6C7). ***Stipe*** 4.5–6 × 0.7–1.2 cm, cylindrical, dirty white (1A1–1B1) and pale violaceous (16A2–16A4), with more and more violaceous (16A4) tint towards the stipe apex when young, later dirty white (1A1–1B1), pale brown (6B2–6B4), covered with brown (6C6) to dark brown (6D6) fibrillose squamules; annulus cortinate; context of stipe dirty white (1A1–1B1) with brown (6C6); basal mycelium white (1A1).

***Basidiospores*** [60/3/3] (10–)11.5–12.5(–14) × (5–)7.5–10 μm, Q = 1.25–1.43(–1.66), av. = 12.06 ± 0.85 × 8.33 ± 1.48 μm, Qav. = 1.48 ± 0.24, ellipsoid to amygdaliform, moderately to strongly verrucose, inamyloid. ***Basidia*** 37.5–43 × 7.5–10 μm, 4-spored, clavate. ***Trama of lamellae*** regular, composed of colorless to yellowish, smooth hyphae 12.5–15 μm wide. ***Cystidia*** absent. ***Pileipellis*** duplex: epicutis weakly developed, 15–20 μm thick, composed of only 2–3 layers of interwoven to parallel, colorless, smooth, thin-walled, long-celled hyphae 3–7.5 µm wide; hypocutis composed of interwoven to parallel, colorless, cylindrical, thin-walled hyphae 12.5–17.5 μm wide. ***Clamp connections*** common in all parts of basidioma.

##### Habitat/host.

Summer. Solitary or gregarious on soil in subalpine temperate broad-leaved and coniferous mixed forests with trees of *Quercus* and *Pinus*.

##### Distribution.

Currently known from southwestern China.

##### Additional specimens examined.

China • Yunnan Province: Lijiang City, Yulong Naxi Autonomous County, Lijiang Alpine Botanical Garden, in a subalpine temperate broad-leaved and coniferous mixed forest with trees of *Quercus* and *Pinus*, 27°0.21'N, 100°10.71'E, elevation 3,340 m, 7 August 2023, Guan-Rui Li 328 (KUN-HKAS 145314), same place and date, Guan-Rui Li 333 (KUN-HKAS 145315).

##### Notes.

*Cortinariuscoriaceus* is characterized by its brown, leathery-wrinkled pileus, pinkish-tinted lamellae, and relatively larger basidiospores.

*Cortinariuscoriaceus* is phylogenetically closely related to and morphologically similar to *C.odoritraganus*, known from Eastern North America and Costa Rica, in mixed temperate forest with *Abies* and *Betula* or mountain *Quercus* forest. However, *C.odoritraganus* differs in its paler pileus, adnexed, purple-brown to brown lamellae, longer and thicker stipe (5–10 × 1–2 cm), and relatively smaller basidiospores (9.5–11.5 × 6–7.5 μm) (Niskanen 2020). *Cortinariusniveotraganus* Kytöv., Niskanen & Liimat., another related species, is distinguished by its hemispherical to broadly convex pileus, initially white to greyish white lamellae with bluish tints, clavate stipe, relatively smaller basidiospores (8.6–10.9 × 5.2–6.3 μm), and occurrence in planted *Betula* forests (Niskanen 2014).

#### ﻿ Cortinarius fuscocandidus

Taxon classificationFungiAgaricalesCortinariaceae

Zhu L. Yang, Liu K. Jia & Zi R. Wang sp. nov.

8D0A656B-CD20-58A6-9921-209107839391

 857352

Fig. 4

##### Etymology.

The epithet “*fuscocandidus*” (Lat.) refers to the dark brown pileus with a white margin of this species.

**Figure 4.**
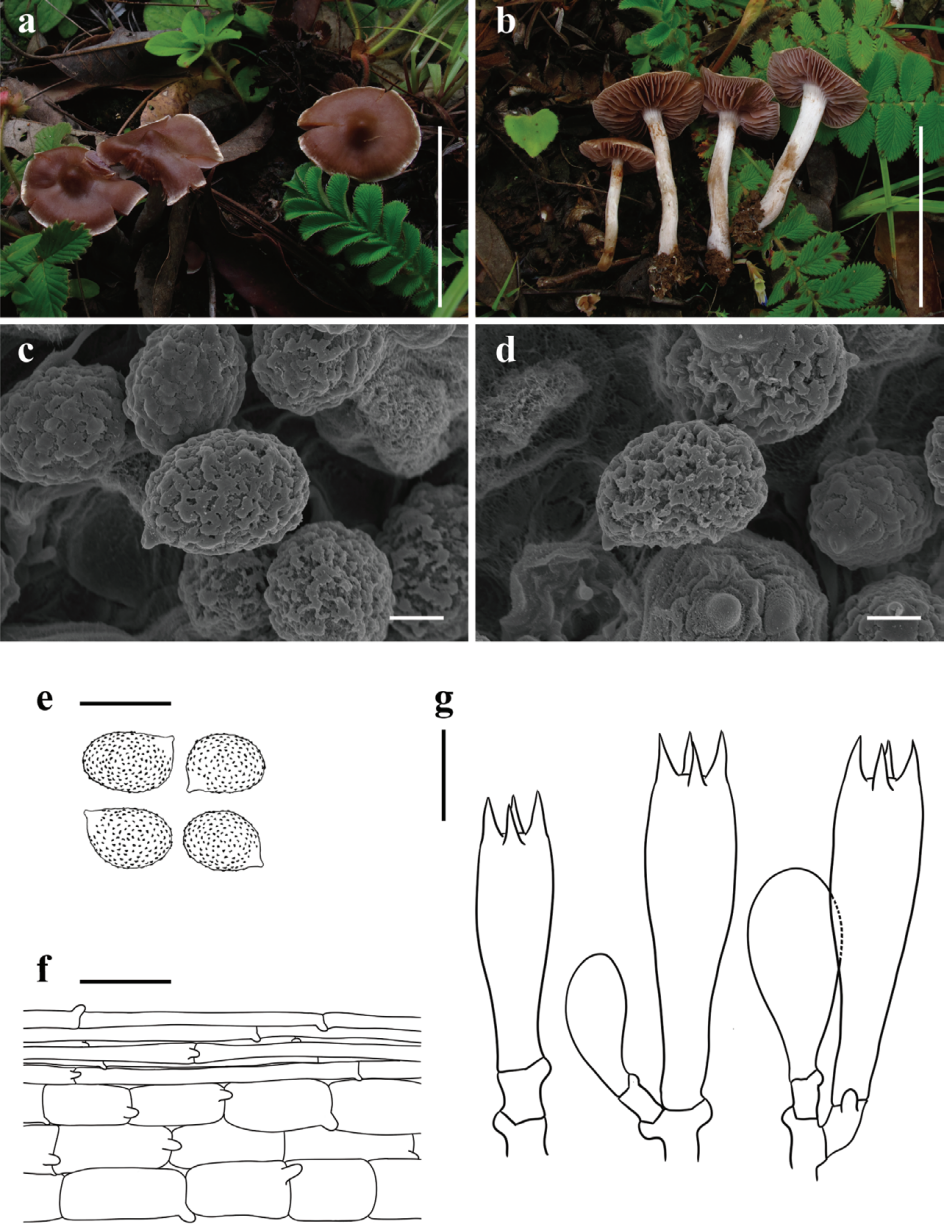
*Cortinariusfuscocandidus* (**a–g**KUN-HKAS 70198, Holotype) **a, b** basidiomata **c–e** basidiospores **f** pileipellis **g** basidia; and marginal sterile cells. Scale bars: 5 cm (**a, b**); 2 μm (**c, d**); 10 μm (**e**); 20 μm (**f, g**).

##### Holotype.

China • Yunnan Province: Lijiang City, Ninglang Yi Autonomous County, Xinyingpan Township, in a subalpine temperate broad-leaved and coniferous mixed forest with trees of *Quercus* and *Pinus*, 27°9.9'N, 100°55.63'E, elevation 2,700 m, 7 August 2011, Qing Cai 602 (KUN-HKAS 70198). GenBank: ITS: PQ772210, nrLSU: PQ772222.

##### Diagnosis.

*Cortinariusfuscocandidus* resembles *C.fulvopaludosus* Kytov., Niskanen & Liimat. (Liimatainen 2017), but differs in its white margin, more robust stipe, and broadly ellipsoid to amygdaliform basidiospores.

##### Description.

***Basidioma*** small. ***Pileus*** 1.8–2 cm diam, applanate to plano-convex with a papilla, viscid; dark brown (6E7); margin white (1A1), sparsely covered with brown (6C6) fibrillose squamules; context of pileus brown (6D7). ***Lamellae*** emarginate with decurrent tooth, medium-spaced (L = 25–33, l = 9–12), pale brown (6B4) with a somewhat pale violaceous (16A2) tint. ***Stipe*** 5–7 × 0.3–0.6 cm, cylindrical, white (1A1) with a somewhat pale violaceous (16A2) tint, pale brown (6B2–6B4) when damaged; annulus cortinate; context of stipe not observed; basal mycelium white (1A1) with a somewhat pale violaceous (16A2) tint.

***Basidiospores*** [60/2/2] 7.5–10.5 × (5–)7–10 μm, Q = 1.07–1.5(–1.65), av. = 8.19 ± 1.24 × 6.99 ± 1.26 μm, Qav. = 1.29 ± 0.18, broadly ellipsoid to amygdaliform, occasionally subglobose, strongly verrucose, inamyloid. ***Basidia*** 20–22.5 × 7.5–10 μm, 4-spored, clavate. ***Trama of lamellae*** regular, composed of colorless, smooth hyphae 7.5–10 μm wide. ***Cystidia*** absent. ***Pileipellis*** duplex: epicutis weakly developed, 11–15 μm thick, gelatinous, composed of interwoven to parallel, colorless, smooth, thin-walled, long-celled hyphae 2.5–5 µm wide, with brownish incrustation; hypocutis composed of only 3–5 layers of interwoven to parallel, colorless, cylindrical, thin-walled hyphae 7.5–15 μm wide. ***Clamp connections*** common in all parts of basidioma.

##### Habitat/host.

Summer. Gregarious on soil in subalpine temperate broad-leaved and coniferous mixed forests with trees of *Quercus* and *Pinus*.

##### Distribution.

Currently known from southwestern China.

##### Additional specimen examined.

China • Yunnan Province: Lijiang City, Gucheng District, Jinshan Township, in a subalpine temperate broad-leaved and coniferous mixed forest with trees of *Quercus* and *Pinus*, 26°54.55'N, 100°18.44'E, elevation 2,145 m, 28 July 2011, Li-Ping Tang 1331 (KUN-HKAS 69792).

##### Notes.

*Cortinariusfuscocandidus* is characterized by its dark brown, papillate pileus with a white margin, pale brown lamellae with a somewhat pale violaceous tint, and broadly ellipsoid basidiospores.

Phylogenetically, *C.fuscocandidus* belongs to sect. Hinnulei and is closely related to *C.fulvopaludosus*. However, the phylogenetic tree shows low support between these two similar species, which can only be distinguished by their margin coloration and basidiospore size (Liimatainen 2017).

Morphologically, *C.fuscocandidus* looks like a typical member of sect. Hinnulei (Fries 1838; Bidaud et al. 2012; Li et al. 2016; Liimatainen et al. 2017; 2020), where the overall coloration of the pileus is brown to dark brown. However, the white margin, somewhat pale violaceous lamellae, and broadly ellipsoid basidiospores (7.5–10.5 × (5–)7–10 μm) differentiate it from the most similar species, *C.badioflavidus* Ammirati et al., which has brown to rich brown lamellae and narrower basidiospores (8.1–10.5 × 5.8–6.5 μm) (Li et al. 2016).

#### ﻿ Cortinarius neodisjungendus

Taxon classificationFungiAgaricalesCortinariaceae

Zhu L. Yang, Liu K. Jia & Zi R. Wang sp. nov.

0FAF6280-53FC-55B5-B039-4E61CC56BC25

 857353

Fig. 5

##### Etymology.

The epithet “*neodisjungendus*” (Lat.) refers to its similarity to *C.disjungendus*.

**Figure 5.**
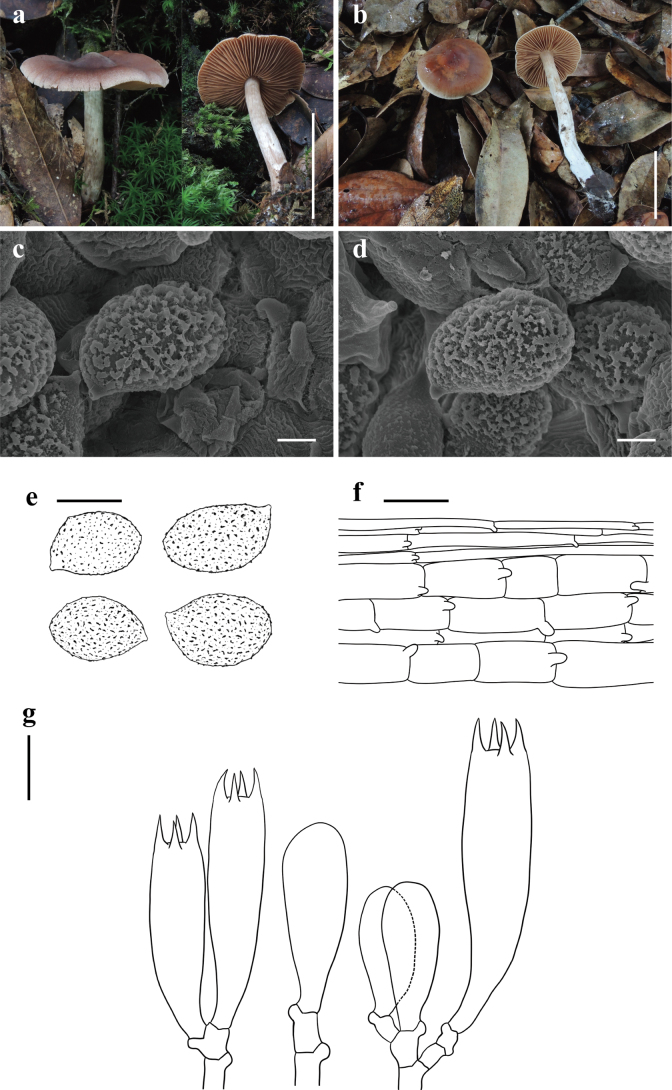
*Cortinariusneodisjungendus* (**a, c–g**KUN-HKAS 145322, Holotype **b**KUN-HKAS 145323) **a, b** basidiomata **c–e** basidiospores **f** pileipellis **g** basidia; and marginal sterile cells. Scale bars: 5 cm (**a, b**); 2 μm (**c, d**); 10 μm (**e**); 20 μm (**f, g**).

##### Holotype.

China • Yunnan Province: Pu’er City, Jingdong Yi Autonomous County Ailao Mountain Subtropical Forest Ecosystem Research Station, Chinese Academy of Sciences, in a subtropical broad-leaved forest with trees of *Quercus*, 24°32.57'N, 101°1.62'E, elevation 2,532 m, 8 October 2021, Jian-Wei Liu 2505 (KUN-HKAS 145322). GenBank: ITS: PQ772207, nrLSU: PQ772219.

##### Diagnosis.

*Cortinariusneodisjungendus* differs from other species within sect. Disjungendi by its plano-convex pileus with an umbo, pale brown coloration, and relatively larger basidiospores (Karsten 1893; Niskanen 2014; Liimatainen et al. 2015).

##### Description.

***Basidioma*** medium-sized. ***Pileus*** 3.5–4.2 cm diam, applanate to plano-convex with an umbonate center, viscid with hygrophanous streaks; pale brown to brown (6D3–6D4), dark brown (6E6) towards the center, white (1A1) to pale brown (6B2) towards the margin, sparsely covered with white (1A1) fibrillose squamules; context not observed. ***Lamellae*** emarginate, crowded (L = 52–61, l = 48–53), pale brown (6B4) to brown (6D6). ***Stipe*** 8–10 × 0.5–0.8 cm, cylindrical with a subbulbous base 1–1.5 cm wide, white (1A1) to pale brown (6B2–6B4), base sparsely covered with brown (6C5) fibrillose squamules; basal mycelium white (1A1).

***Basidiospores*** [60/2/2] 11–13.5(–15) × (5–)7.5–9 μm, Q = 1.43–1.71(–2), av. = 12.73 ± 0.93 × 7.52 ± 0.96 μm, Qav. = 1.71 ± 0.2, broadly amygdaliform, strongly verrucose, inamyloid. ***Basidia*** 32.5–40 × 7.5–10 μm, 4-spored, clavate. ***Trama of lamellae*** regular, composed of colorless to brownish, smooth hyphae 10–12.5 μm wide. ***Cystidia*** absent. ***Pileipellis*** duplex: epicutis weakly developed, 8.5–15 μm thick, composed of only 3–5 layers of interwoven to parallel, colorless to brownish, smooth, thin-walled, long-celled hyphae 2.5–5 µm wide; hypocutis composed of interwoven to parallel, colorless to pale brownish, cylindrical, thin-walled hyphae 12.5–15 μm wide. ***Clamp connections*** common in all parts of basidioma.

##### Habitat/host.

Autumn. Solitary on soil in subtropical broad-leaved forests with trees of *Quercus*.

##### Distribution.

Currently known from southwestern China.

##### Additional specimen examined.

China • Yunnan Province: Pu’er City, Jingdong Yi Autonomous County Ailao Mountain Subtropical Forest Ecosystem Research Station, Chinese Academy of Sciences, in a subtropical broad-leaved forest with trees of *Quercus*, 24°32.57'N, 101°1.62'E, elevation 2,532 m, 8 October 2021, Jian-Wei Liu 2529 (KUN-HKAS 145323).

##### Notes.

*Cortinariusneodisjungendus* is characterized by its hygrophanous, pale brown to brown pileus with a whitish margin, whitish stipe, and relatively larger basidiospores. All other species in sect. Disjungendi have a brownish pileus lacking a white margin, a brown stipe, and smaller basidiospores (range from 9–11 μm long, 6–7 μm wide) (Karsten 1893; Niskanen 2014; Liimatainen et al. 2015, 2020).

#### ﻿ Cortinarius sinoconfirmatus

Taxon classificationFungiAgaricalesCortinariaceae

Zhu L. Yang, Liu K. Jia & Zi R. Wang sp. nov.

56BE89C3-F542-5832-9D7D-9E2207C0D673

 857354

Fig. 6

##### Etymology.

The epithet “*sinoconfirmatus*” (Lat.) refers to the species in China that is similar to *C.confirmatus*.

**Figure 6.**
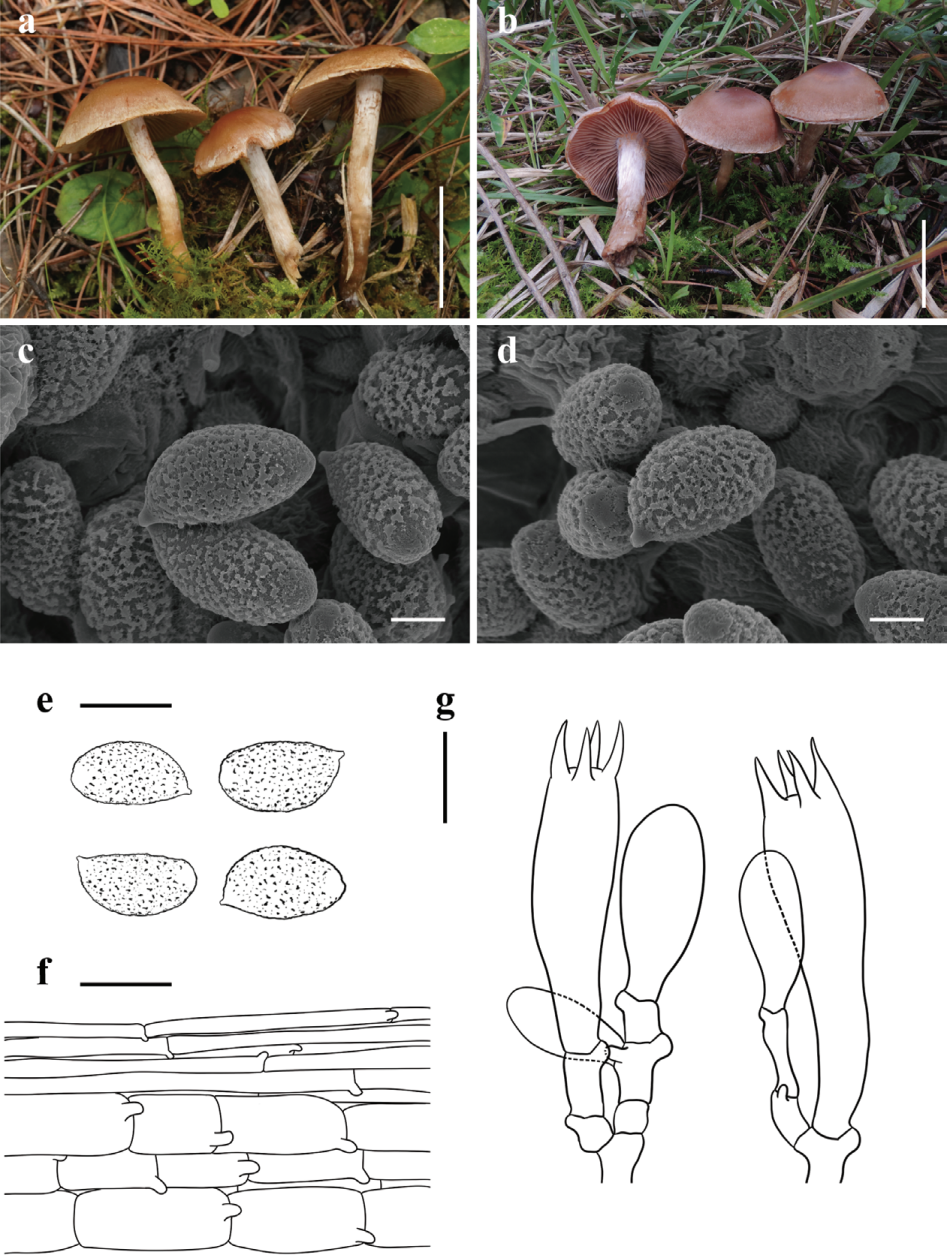
*Cortinariussinoconfirmatus* (**a, c–g**KUN-HKAS 145320, Holotype **b**KUN-HKAS 145318) **a, b** basidiomata **c–e** basidiospores **f** pileipellis **g** basidia; and marginal sterile cells. Scale bars: 5 cm (**a, b**); 2 μm (**c, d**); 10 μm (**e**); 20 μm (**f, g**).

##### Holotype.

China • Yunnan Province: Lijiang City, Yulong Naxi Autonomous County, Taian Township, in a subalpine temperate coniferous forest with trees of *Pinus*, 26°48.91'N, 100°5.96'E, elevation 2,633 m, 9 August 2023, Zi-Rui Wang 160 (KUN-HKAS 145320). GenBank: ITS: PQ772205, nrLSU: PQ772217.

##### Diagnosis.

*Cortinariussinoconfirmatus* looks like *C.confirmatus* Rob. Henry, but differs in its dark brown pileus center, more brown lamellae, thinner stipe, and larger basidiospores (Henry 1983; Mahiques et al. 2001; Ortega et al. 2007; Liimatainen et al. 2017).

##### Description.

***Basidioma*** medium-sized. ***Pileus*** 1.2 cm diam when young, 3–4.3 cm diam when mature, hemispherical when young, later convex, viscid; pale brown (6B2–6B4) to brown (5C6–5C7), covered with white (1A1) fibrillose squamules when young; brown (6C4–6C6), pale brown (6B2–6B4) towards the margin, dark brown (6E7) towards the center when mature; margin covered with brown (6C7) fibrillose squamules; context of pileus gelatinous, pale brown (6B2–6B4) to brown (6C7). ***Lamellae*** emarginate, crowded (L = 74–95, l = 46–52), pale brown (6B2–6B3) with a faint pinkish (12A2) tint when young, later brown (6B6–6C6). ***Stipe*** 5–7 × 0.5–0.7 cm, cylindrical, dirty white (1A1–1B1), pale brown (6B2–6B3) to brown (6C6), with a pale violaceous (16A2–16A3) tint at the stipe apex when young, later the upper 1/2 stipe dirty white, pale brown (6B2–6B3) to brown (6C6) with a pale violaceous (16A2–16A3) tint, covered with brown (7C4) fibrillose squamules, the lower 1/2 brown to dark brown (7B4–7E4); context of stipe dirty white (1A1–1B1) and brown (7C6); basal mycelium white (1A1).

***Basidiospores*** [60/3/3] 7.5–11.5 × 4–5(6) μm, Q = (1.5–)2–3.13, av. = 9.92 ± 1.19 × 4.85 ± 0.59 μm, Qav. = 2.06 ± 0.28, ellipsoid to narrowly ellipsoid, moderately to strongly verrucose, inamyloid. ***Basidia*** 27.5–35 × 5–7.5 μm, 4-spored, clavate. ***Trama of lamellae*** regular, composed of pale yellowish, smooth hyphae 12.5–15 μm wide. ***Cystidia*** absent. ***Pileipellis*** duplex: epicutis weakly developed, 10–14 μm thick, gelatinous, composed of only 2–4 layers of interwoven to parallel, colorless to pale yellow, smooth, thin-walled, long-celled hyphae 2.5–5 µm wide; hypocutis composed of interwoven to parallel, colorless, cylindrical, thin-walled hyphae 12.5–17.5 μm wide. ***Clamp connections*** common in all parts of basidioma.

##### Habitat/host.

Summer. Gregarious on soil in subalpine temperate coniferous forests with trees of *Pinus*.

##### Distribution.

Currently known from southwestern China.

##### Additional specimens examined.

China • Yunnan Province: Lijiang City, Yulong Naxi Autonomous County, Taian Township, in a subalpine temperate coniferous forest with trees of *Pinus*, 26°48.91'N, 100°5.96'E, elevation 2,633 m, 9 August 2023, Zi-Rui Wang 154 (KUN-HKAS 145319); same Township and date, 26°48.32'N, 100°4.35'E, elevation 2,700 m, Dong-Mei Li 331 (KUN-HKAS 145318).

##### Notes.

*Cortinariussinoconfirmatus* is closely related to *C.confirmatus*, but the latter differs from the former by its paler pileus with vinaceous or violaceous tints, paler, adnate lamellae, more robust stipe, and narrower basidiospores (8.8–10 × 5.2–5.6 μm, Q = 1.55–1.9) (Henry 1983; Mahiques et al. 2001; Ortega et al. 2007; Liimatainen et al. 2017). *Cortinariussinoconfirmatus* is also closely related to *C.imbutus* Fr. and *C.saturninus* (Fr.) Fr. However, *C.imbutus* differs from *C.sinoconfirmatus* by its pale yellow pileus and whitish stipe with somewhat violaceous tint at the stipe apex (Fries 1838), and *C.saturninus* differs from *C.sinoconfirmatus* by its dark reddish brown pileus, violet stipe with purplish red squamules (Fries 1838).

Morphologically, *C.sinoconfirmatus* looks like *C.lucorum* (Fr.) E. Berger, but the latter differs from the former by its pileus with marble-like stripes and more prominent bulbous stipe base (Bidaud et al. 2000; Matheny and Ammirati 2006).

## ﻿Discussion

### ﻿Phylogenetics of five new species within *Cortinarius*

In this study, five species of *Cortinarius* are described as new to science based on phylogenetic evidence and morphological characteristics. Our phylogenetic tree reveals that four of these species—*C.coriaceus*, *C.fuscocandidus*, *C.neodisjungendus*, and *C.sinoconfirmatus*—belong to subgen. Telamonia, while the relationships between *C.coriaceus* and *C.niveotraganus*, as well as *C.sinoconfirmatus* and *C.confirmatus*, have been resolved (Fig. 1). The phylogenetic position of *C.fuscocandidus* remains uncertain. Additionally, *C.brunneoverrucosus* is assigned to sect. Dulciolentes (Fig. 1), a small section not yet placed in any subgenus of *Cortinarius* (Liimatainen et al. 2022). *Cortinariusneodisjungendus* forms a strongly sister clade (98.8/99/1.00) with other species within sect. Disjungendi, but differs by its whitish margin and whitish stipe (Karsten 1893; Liimatainen et al. 2015, 2020).

### ﻿Ecological distribution of five new species within *Cortinarius*

Ecologically, the five species fall into two categories: *Cortinariuscoriaceus*, *C.fuscocandidus*, and *C.sinoconfirmatus* inhabit subalpine temperate areas, whereas *C.brunneoverrucosus* and *C.neodisjungendus* are restricted to subtropical areas. Notably, within sect. Dulciolentes, three sequestrate species—*C.peraurantiacus*, *C.pisciodorus*, and *C.dulciolens*—are known only from Oceania, while the agaricoid *C.corrugatus* occurs in North America (Peck 1872; Moser 1983; Peintner et al. 2002a, 2002b; Phillips 2010; Soop et al. 2019; Kuo 2020). The discovery of *C.brunneoverrucosus* in China represents the first record of sect. Dulciolentes in East Asia. Furthermore, the agaricoid basidioma of *C.brunneoverrucosus* provides evidence of biogeographic linkages between North America and East Asia.

## Supplementary Material

XML Treatment for Cortinarius brunneoverrucosus

XML Treatment for Cortinarius coriaceus

XML Treatment for Cortinarius fuscocandidus

XML Treatment for Cortinarius neodisjungendus

XML Treatment for Cortinarius sinoconfirmatus

## ﻿Additional information

### Author ORCIDs

Liu-Kun Jia https://orcid.org/0000-0002-3180-5077

Zi-Rui Wang https://orcid.org/0000-0003-4564-1904

Zhu-Liang Yang https://orcid.org/0000-0001-9745-8453

### Data availability

Sequence alignments were deposited in TreeBASE (Study ID: TB2:S31924; URL: http://purl.org/phylo/treebase/phylows/study/TB2:S31924). DNA sequences (Table 2) are available in GenBank (https://www.ncbi.nlm.nih.gov/genbank/).
